# IL-10 Protects Neurites in Oxygen-Glucose-Deprived Cortical Neurons through the PI3K/Akt Pathway

**DOI:** 10.1371/journal.pone.0136959

**Published:** 2015-09-14

**Authors:** Longzai Lin, Hongbin Chen, Yixian Zhang, Wei Lin, Yong Liu, Tin Li, Yongping Zeng, Jianhao Chen, Houwei Du, Ronghua Chen, Yi Tan, Nan Liu

**Affiliations:** 1 Department of Neurology, The Affiliated Union Hospital, Fujian Medical University, Fuzhou, Fujian, People’s Republic of China; 2 Institute of Cerebral Vascular Disease of Fujian Province, Fuzhou, Fujian, People’s Republic of China; 3 Department of Rehabilitation, The Affiliated Union Hospital, Fujian Medical University, Fuzhou, Fujian, People’s Republic of China; School of Pharmacy, Texas Tech University HSC, UNITED STATES

## Abstract

IL-10, as a cytokine, has an anti-inflammatory cascade following various injuries, but it remains blurred whether IL-10 protects neurites of cortical neurons after oxygen-glucose deprivation injury. Here, we reported that IL-10, in a concentration-dependent manner, reduced neuronal apoptosis and increased neuronal survival in oxygen-glucose-deprived primary cortical neurons, producing an optimal protective effect at 20ng/ml. After staining NF-H and GAP-43, we found that IL-10 significantly protected neurites in terms of axon length and dendrite number by confocal microscopy. Furthermore, it induced the phosphorylation of AKT, suppressed the activation of caspase-3, and up-regulated the protein expression of GAP-43. In contrast, LY294002, a specific inhibitor of PI3K/AKT, reduced the level of AKT phosphorylation and GAP-43 expression, increased active caspase-3 expression and thus significantly weakened IL-10-mediated protective effect in the OGD-induced injury model. IL-10NA, the IL-10 neutralizing antibody, reduced the level of p-PI3K phosphorylation and increased the expression of active caspase-3. These findings suggest that IL-10 provides neuroprotective effects by protecting neurites through PI3K/AKT signaling pathway in oxygen-glucose-deprived primary cortical neurons.

## Introduction

Inflammation is a key pathobiological element of stroke, from early acute injury to late neuron remodeling and function reconstruction and anti-inflammation has been demonstrated to reduce effectively both cerebral infarct area in vivo and neuron death in vitro [1,2]. As an anti-inflammatory cytokine, IL-10 can suppress the inflammatory cascade following ischemic brain injury, which has been confirmed in the animal model of ischemic stroke using exogenous IL-10, IL-10-overexpressing transgenic mice and IL-10-expressing virus-vector [3–5]. It has been demonstrated to reduce neuronal apoptosis in vitro after exposing the primary cortical neurons to oxygen-glucose deprivation (OGD) and activate the PI3K/AKT pathways, which further triggers the canonical NF-κB pathway [6,7]. As transcriptional regulators, NF-κB proteins promote expression of gene products and modulate neuronal differentiation, neurite extension, dendritic plasticity [8,9]. However, little direct evidence is available to document that IL-10 protects neurites after neuron injury. In the present study, we found that IL-10 not only improved the viability of primary cortical neurons but also protected neurites in the OGD-induced neuron injury model in vitro.

## Methods

### Ethics Statement

All animals were provided by Animal Center of Fujian Medical University (Fuzhou, China). Animals used in this study were cared for in accordance with the National Institute of Health Guide for the Care and Use of Laboratory Animals (NIH Publications No. 80–23, revised in 1996). All procedures were approved by Institutional Animal Care and Use Committee of Fujian Medical University. Efforts were made to minimize the number of animals used as well as their suffering.

### Primary Cultures of Cortical Neurons

Primarily cultured cortical neurons were isolated as described previously with slight modification [10,11]. Briefly, samples of cerebral cortex were obtained from pregnant Sprague-Dawley rats (16–18 days old). All animals were euthanized with isoflurane (3% induction, 1.5% maintenance in 30% O_2_ and 70% N_2_O). The cell sediment was resuspended in the neurobasal medium (Gibco, USA) containing 2% B27 (Gibco, USA), 0.5 mM of glutamine, and 50 U/ml of penicillin/streptomycin. This neurobasal medium contained approximately 90% neurons as determined by class III-β-Tubulin and Hoechst 33342 staining.

### Oxygen-Glucose Deprivation

Oxygen glucose deprivation (OGD) model was established as previously described with slight modification [12]. Briefly, five days after culture, the primary cortical neurons were washed with glucose-free DMEM (Gibco, USA). Then, after the addition of glucose-free DMEM, they were placed in an anaerobic chamber containing 5% CO_2_ and 95% N_2_ at 37°C. OGD was terminated after 1.5h by replacing the glucose-free DMEM with original medium and the neurons were further incubated in a chamber containing 95% air and 5% CO_2_ at 37°C for 24h or 48h. Drugs were added into the medium right before the onset of OGD. Cells in the control group were treated without the OGD exposure.

### Drug Treatment

IL-10 was dissolved in double distilled water and the final concentration in the medium was 20ng/ml. LY294002 (20umol/L, a specific inhibitor of PI3K/AKT) was added to the cells with or without IL-10 for 24h after OGD by flow cytometry to detect neuron apoptosis or 48h after by immunofluorescence to measure the length and the number of the neurites. The groups were designed as follows: control group, IL-10 (20ng/ml)+OGD group, IL-10 (20ng/ml)+LY294002 (20umol/L)+OGD group, LY294002 (20umol/L)+OGD group, OGD group. In order to better understand the mechanisms underlying the neuroprotective effects of IL-10, a further experiment with a similar design was performed with a different agent, IL-10NA, the IL-10 neutralizing antibody (Abcam). IL-10NA (5μg/mL) was administered to cultured neurons after OGD, as previously reported [13,14], and the designed groups were: Control group, IL-10 (20ng/ml) +OGD group, IL-10NA (5μg/mL) +OGD group, OGD group. All the groups of these experiments were given equal volumes of medium.

### Flow Cytometry Using Annexin V/PI Staining

To detect the neuron apoptosis, we performed flow cytometry as previously described with slight modification [15]. In brief, cells were seeded in 25 cm^2^ culture flasks according to the above experimental protocol. Twenty-four hours after OGD, the Annexin V/PI (Beyotime, China) staining was performed according to the manufacturer’s instruction. Before flow-cytometric analysis, 10μl of Annexin-V-FITC labeling reagent and 10μl of PI were added to the medium and the neurons were incubated at room temperature for 15 min in the dark (n = 3 for each group). Each sample contained 1× 10 ^5 cells and was analyzed immediately.

### Western Blotting

After the treatment, primary cortical neurons were washed and lysed with RIPA lysis buffer (Beyotime, China). The solution was centrifuged at 14000 x g at 4°C for 10min. Concentration of the protein was measured by the BCA protein assay (Beyotime, China). Extracts were separated by 10% or 12% sodium dodecyl sulfate-polyacrylamide gel electrophoresis (SDS-PAGE) and transferred to PVDF membranes (Millipore, USA). Membranes were probed with primary antibodies at 4°C overnight (anti-pPI3K and anti-PI3K, 1:1000, Abcam; anti-pAKT and anti-AKT, 1:1000, Cell Signaling Technology; anti-GAP-43, 1:1000, Cell Signaling Technology; anti-GAPDH, 1:500, Boster; anti-activated-caspase-3, 1:500, Beyotime). Band intensities were analyzed with the image J software (1.46r).

### Immunofluorescence Staining and Neurites Assay

Immunofluorescence was performed to determine the changes of neurites and nuclei as previously described [10,16]. Forty-eight hours after OGD injury, fixed in 4% paraformaldehyde (pH 7.4), cells were incubated with primary antibodies at 4°C overnight (rabbit anti-GAP-43 monoclonal antibody, 1:200, Cell Signaling Technology; rabbit anti-activated-caspase-3, 1:200, Beyotime; mouse anti-class III-β-Tubulin monoclonal antibody 1:200, Beyotime). After washing, they were further incubated with corresponding secondary antibodies (Cy3 donkey anti-mouse IgG, 1:400, Jackson Immunoresearch; Dylight488 donkey anti-rabbit IgG, 1:400, Jackson Immunoresearch). Nuclei were stained with Hoechst33342 (Sigma, USA). Glass slides were detected with a ZEISS LSM 710 confocal microscope (Carl Zeiss, Germany), and the length of axon and the number of dendrites were analyzed with LSM Image Browser (V4.2.0.121). Meanwhile, 24 hours after OGD injury, the number of apoptotic cells was calculated. For each group and experiment, 3 visual fields in every coverslip were observed and all trials were repeated three times.

### Statistical Analysis

Data were expressed as Mean ± SD and analyzed by SPSS16.0 statistical software (IBM, USA). Each procedure was carried out in replication in 3–5 independent trials. Statistical significance was determined by one way analysis of variance (ANOVA) and LSD’s multiple comparisons test. Dunnett’s T3 was applied when equal variances were not assumed. *P* value less than 0.05 (two-sided) was considered statistically significant.

## Results

### IL-10 Reduces the Neuronal Apoptosis and Increases the Survival of Oxygen-Glucose-Deprived Neurons

**Fig 1:**
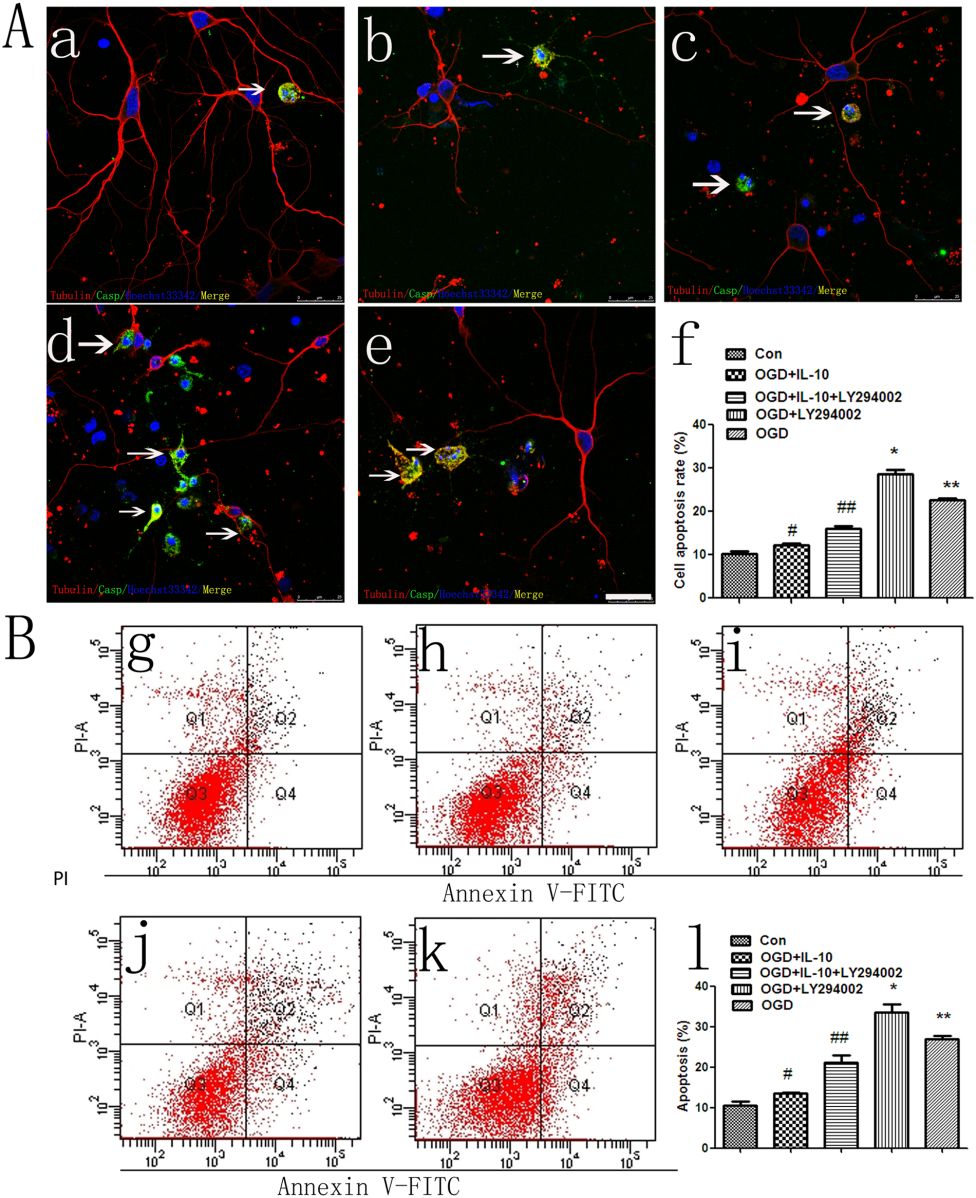
IL-10 reduces the neuronal apoptosis in OGD-induced injury model. **(A)** Caspase-3 was performed to detect the cell apoptosis, showing apoptotic cell (green, arrows). The neurons were stained with class III-β-Tubulin (red). The apoptotic neurons were labeled yellow. (a) Normal control group. (b) OGD + IL-10 (20ng/ml) group. (c) OGD + IL-10 (20ng/ml) + LY294002 (20μM) group. (d) OGD+LY294002 (20μM) group. (e) OGD group. (f) **p<0.01 vs control group, #*p*<0.01 vs OGD group, ##*p*<0.05 vs OGD+IL-10 group,*p<0.01 vs OGD group. Scale bar, 25μm. **(B)** After 24h, assay of apoptosis by flow cytometry. The signals from apoptosis of neurons were localized in the four quadrant except Q3 of the resulting dot-plot graph. (g) Normal control group. (h) OGD + IL-10 (20ng/ml) group. (i) OGD + IL-10 (20ng/ml) + LY294002 (20μM) group. (j) OGD+LY294002 (20μM) group. (k) OGD group. (l) **p<0.01 vs Normal control group,# *p*<0.01 vs OGD group, *p<0.01 vs OGD group.

To study the effect of IL-10 on apoptosis, we treated primary cultured neurons with IL-10 in combination with or without LY294002 in OGD-induced neuron injury model. As indicated in the corresponding figs, the morphology of most nucleuses was round and uniform, while few fragmented and consolidated in the normal control group [Fig 1A-a]. In contrast, most nuclei were consolidated in the OGD group [Fig 1A-e]. Compared with the OGD group and OGD+LY294002 (20umol/L) group, OGD+ IL-10 (20ng/ml) group respectively reported a lower apoptosis rate 24h after OGD injury (12.16 ± 1.69 vs. 22.61 ± 1.35, *p*<0.01; 12.16 ± 1.69 vs. 28.65 ± 3.02, *p*<0.01). Compared with the OGD+IL-10 group, IL-10 with LY294002 induced a higher apoptosis (12.16 ± 1.69 vs. 15.98 ± 1.72, *p*<0.05). There was no significant difference between control group and OGD+IL-10 group (10.31 ± 1.70 vs. 12.16 ± 1.69, *p*>0.05).

Meanwhile, in flow cytometry [Fig 1B], compared with the OGD group and OGD+LY294002 (20umol/L) group, OGD+IL-10 (20ng/ml) group respectively reported a lower apoptosis rate 24h after OGD injury (13.43 ±0.65 vs. 26.93±1.53, *p*<0.01; 13.43 ±0.65 vs. 33.53 ± 3.56, *p*<0.01). Compared with the OGD+IL-10 group, IL-10 with LY294002 induced a higher apoptosis (13.43 ±0.65 vs. 21.17 ± 2.97, *p*<0.01). There was no significant difference between control group and OGD+IL-10 group (10.47 ± 1.94 vs. 13.43 ±0.65, *p*>0.05).

### IL-10 Protects the Neurites from OGD Injury

**Fig 2:**
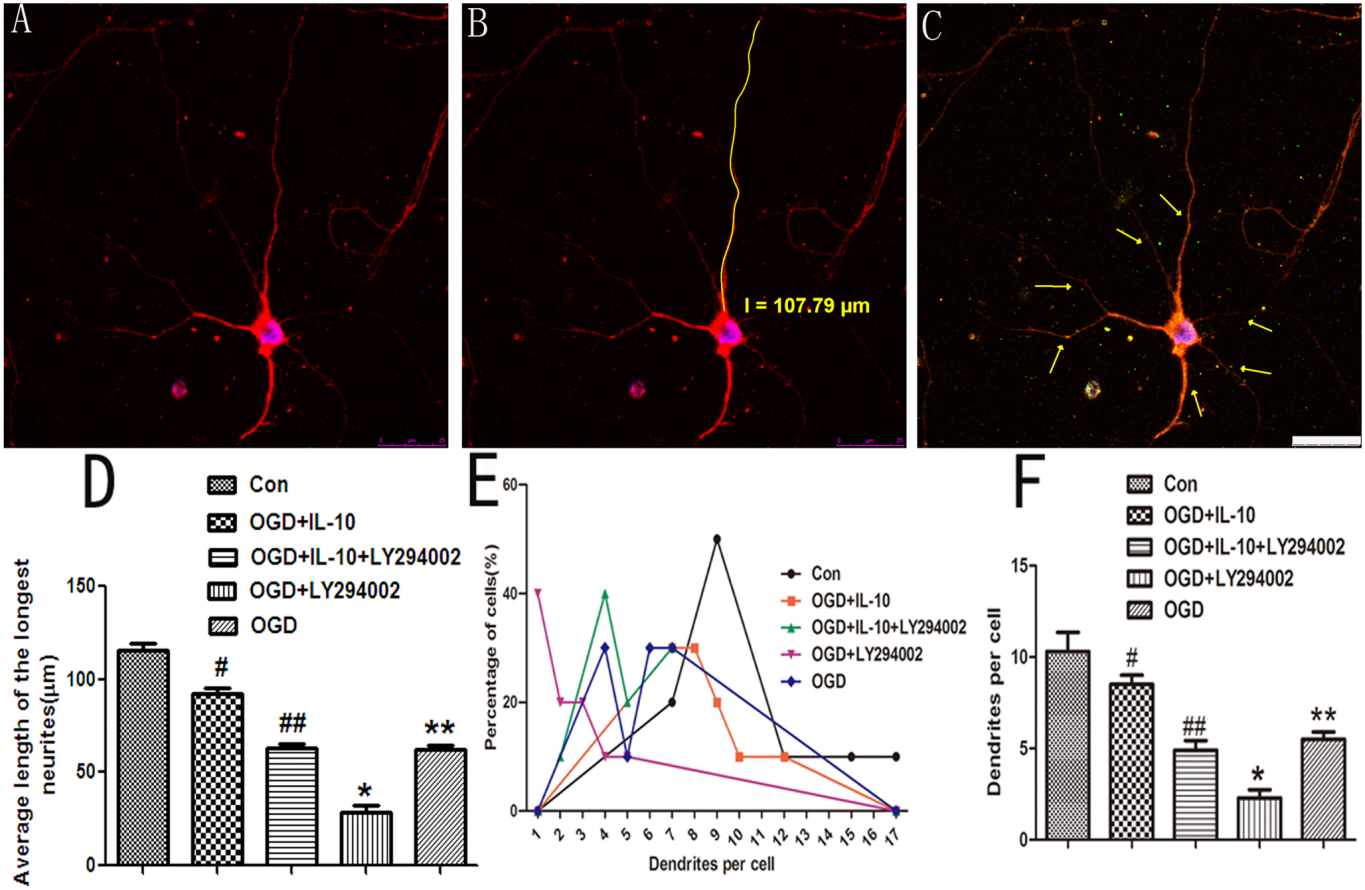
Detection of the axons and dendrites by laser confocal microscopy. (A) neuron body, cell dendrite (red) and nucleus (blue) were shown. (B) Manually tracing length of the axon (yellow) by the LSM (4.2.0.121) software. (C) Dendrites of neuron (arrows). (D) Quantification of the longest neurites. Data are expressed as mean ± SD (n = 9). **p<0.01 vs CON, #*p*<0.01 vs OGD group, ##*p*<0.01 vs OGD+IL-10 group,#*p*<0.01 vs Control group, *p<0.01 vs OGD group. (E) Frequency distributions of the number of dendrites per projection in each group. (F) Quantification of the maximum dendrites number of neurons. Values correspond to mean± SD (n = 10). #*p* < 0.01 vs OGD group, *p < 0.01 vs OGD group. Scale bar: 25μm.

**Fig 3:**
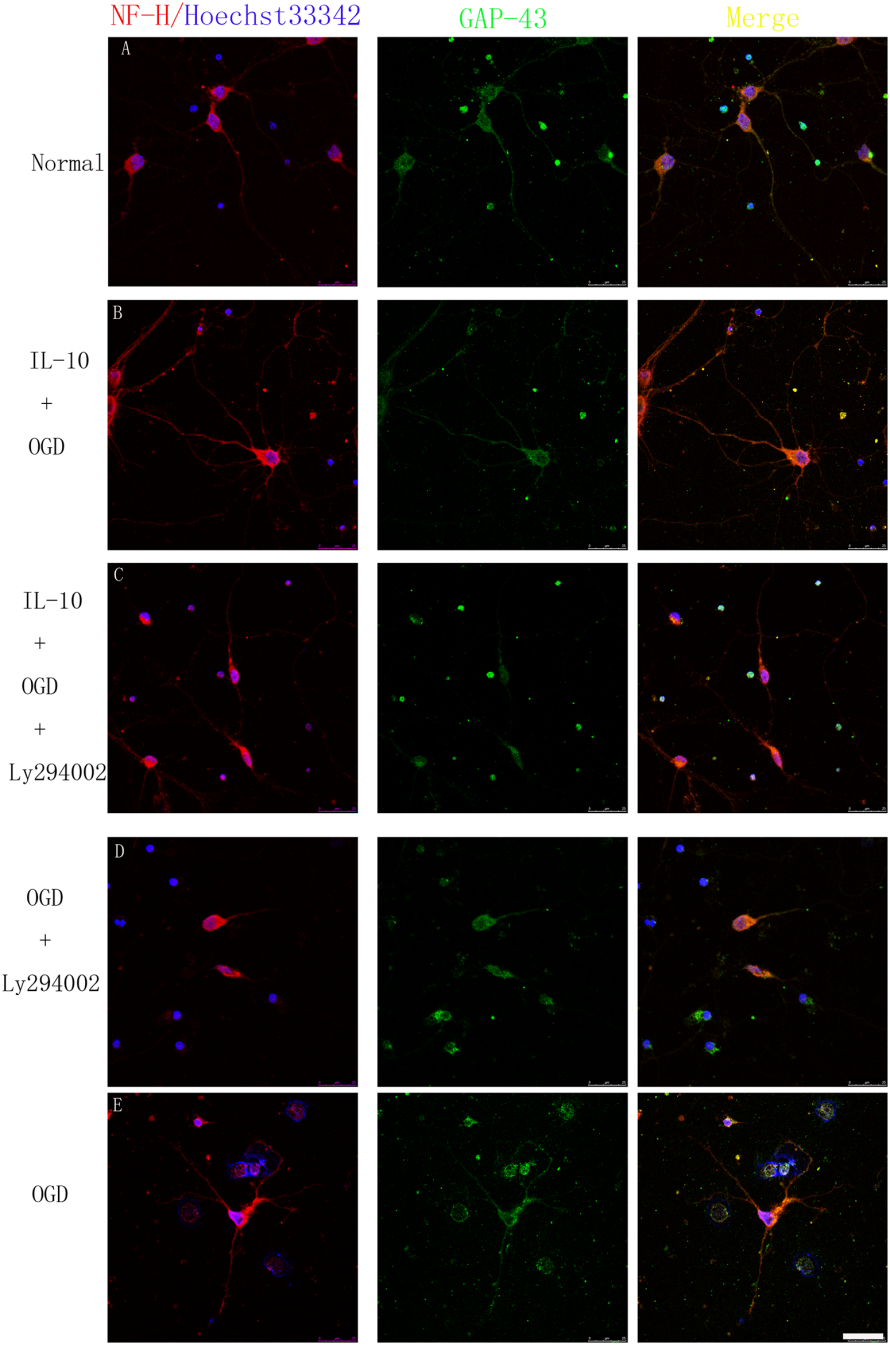
Effect of IL-10 on axons and dendrites. The left column displays neuronal marker NF-H (red) and nucleus (blue). The length of the axons and the numbers of the dendrites were clearly shown. The middle column displays the expression of GAP-43 (green), which is expressed in the somas, axons and dendrites. The right column shows GAP-43 mainly located in the proximal axons (yellow).

To further investigate the direct effect of IL-10 on neurites, we treated oxygen-glucose-deprived neurons with IL-10 in combination with or without LY294002. As shown in Figs 2 and 3, we found that some axons were fractured and shortened after OGD treatment. When compared with that of OGD group, the average length of axon was decreased in OGD+LY294002 group (28.97±9.88μm vs. 62.53±6.44μm, *p*<0.01), and was increased in OGD+IL-10 group (92.70±8.05μm vs. 62.53±6.44μm, *p*<0.01). There was no significant difference between OGD+IL-10+LY294002 and OGD group (62.77±8.49μm vs. 62.53±6.44μm, *p*>0.05). Compared with OGD group, the average number of dendrites was decreased in OGD+LY294002 group (*p*<0.01) and increased in OGD+IL-10 group (*p*<0.01). In OGD+LY294002 group, some neurons lost its dendrites after OGD injury (Fig 3E). There was no significant difference between OGD+IL-10 and normal control group (*p*>0.05) and between OGD+IL-10+LY294002 and OGD group (*p*>0.05). As shown in Fig 2E, the curve peak of the OGD group was shifted to the left when compared with that of the control group, which indicates the serious cell damage in the OGD group. The peak of the OGD+IL-10 group (red line) approximated that of the control group (black line). These data suggest that IL-10 protects neurites from OGD-induced injury and that these protective effects can be partly neutralized by the PI-3K inhibitor LY294002.

### IL-10 Decreases the Expression of the Active Caspase-3 and Increases the Expression of p-AKT and GAP-43

**Fig 4:**
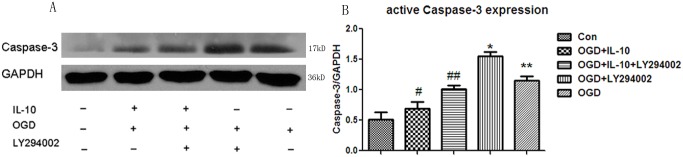
Detection of active Caspase-3 expression by western blot. (A) The most representative image of western blot analysis for active Caspase-3 expression. (B) Statistical graph of active Caspase-3 expression in different groups (n = 3) **p<0.01 vs Control group, #*p*<0.01 vs OGD group, *p<0.01 vs OGD group.

**Fig 5:**
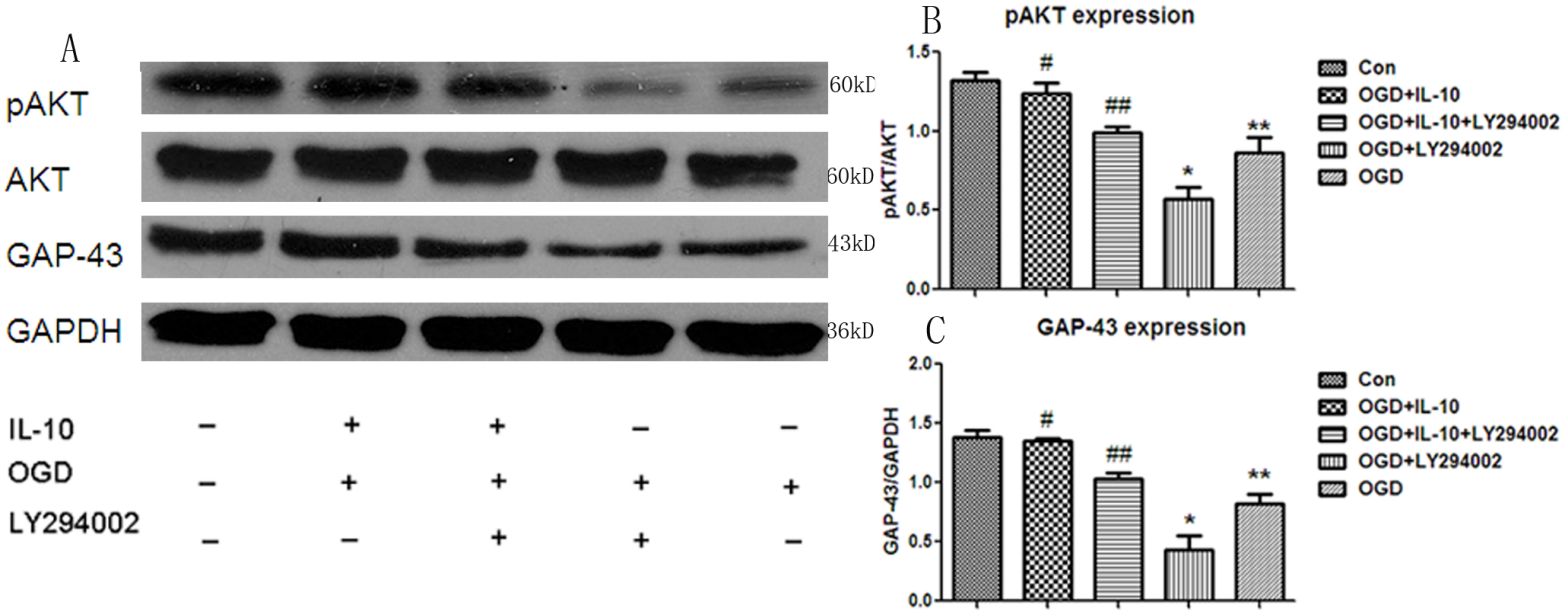
Detection of p-AKT and GAP-43 expression by western blot. (A) The representative image of western blot analysis for p-AKT and GAP-43 expression. (B) Statistical graph of p-AKT expression in different groups (n = 3). #*p*<0.01 vs OGD group; *p<0.05 vs OGD group; **p<0.01 vs Control group. (C) Statistical graph of GAP-43 expression in different groups (n = 3), #*p*<0.01 vs OGD group, *p<0.01 vs OGD group, **p<0.01 vs Control group, ##*p*>0.05 vs OGD group.

To explore the mechanisms of IL-10’s neuroprotective effects, we next analyzed the expression levels of the pro-apoptotic protein active caspase-3, GAP-43 and p-AKT by western blot. Compared with that of the OGD group, the expression of active caspase-3 was increased in the OGD+LY294002 group (*p*<0.05) but significantly decreased in the OGD+IL-10 group (*p*<0.01) (Fig 4). Compared with that of the OGD+IL-10 group, the expression of active caspase-3 in the OGD+IL-10+LY294002 group was significantly increased (*p*<0.05). There was no significant difference between the OGD+IL-10 group and normal control group (*p*>0.05) and between the OGD+IL-10+LY294002 group and OGD group (*p*>0.05). Compared with that of the OGD group, the expression of p-AKT and GAP-43 was decreased in the OGD+LY294002 group but significantly increased in the OGD+IL-10 group (*p*<0.05, respectively) (Fig 5). Compared with those of the OGD+IL-10 group, the expressions of p-AKT and GAP-43 in the OGD+ IL-10+LY294002 group were significantly decreased (*p*<0.05). There was no significant difference between the OGD+IL-10 and control group (*p*>0.05) and between the OGD+IL-10+LY294002 and OGD group (*p*>0.05). These data indicate that IL-10 up-regulates the expression of GAP-43 and p-AKT and down-regulates the level of active caspase-3 and that these effects can be partly neutralized by inhibitor LY294002.

### IL-10NA Partly Suppresses the Regulatory Effect of IL-10 on the Expression of p-PI3K, p-AKT, and the Active Caspase-3

**Fig 6:**
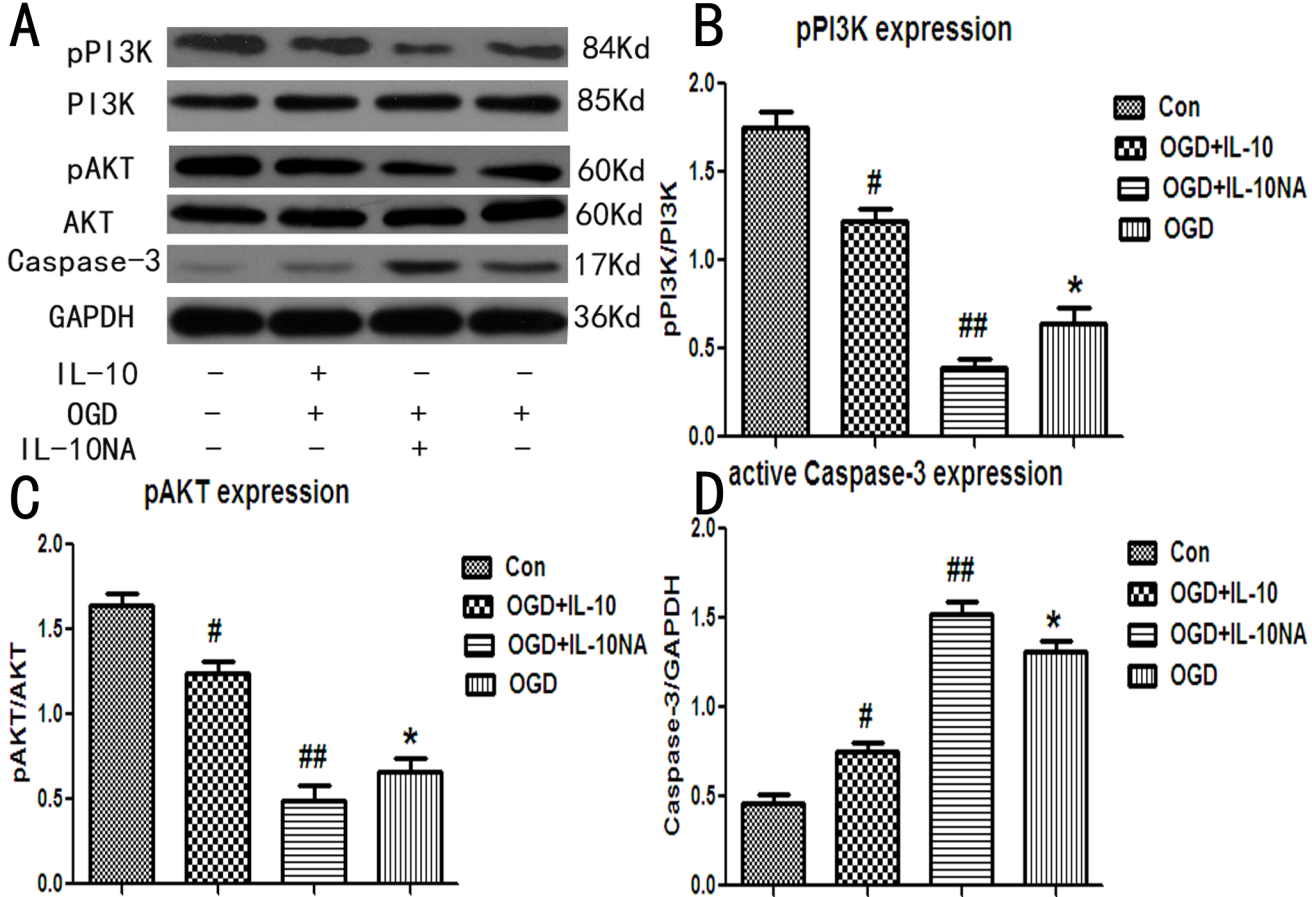
Detection of p-PI3K, p-AKT and active Caspase-3 expression by western blot. (A) The most representative image of western blot analysis for p-PI3K, p-AKT and active Caspase-3. (B) Statistical graph of p-PI3K expression in different groups (n = 3), #*p*<0.01 vs OGD+IL-10NA group, #*p*<0.01 vs OGD group, ##*p*<0.05 vs OGD group. (C) Statistical graph of p-AKT expression in different groups (n = 3), #*p*<0.01 vs OGD+IL-10NA group, #*p*<0.01 vs OGD group, ##*p*>0.05 vs OGD group. (D) Statistical graph of active Caspase-3 expression in different groups (n = 3), #*p*<0.01 vs OGD+IL-10NA group, #*p*<0.01 vs OGD group, ##*p*<0.01 vs OGD group.

To further demonstrate whether IL-10 could protect OGD-injured neurons through the PI3K/AKT signaling pathway, we added IL-10NA to neurons after OGD injury and then examined the expression of p-PI3K, p-AKT and the active caspase-3. Compared with those of the OGD group, the expression of p-PI3K was decreased in the OGD+IL-10NA group (*p*<0.05), but significantly increased in the OGD+IL-10 group (*p*<0.01) (Fig 6B); the expression of p-AKT was increased in the OGD+IL-10 group (*p*<0.01), but no significant difference was found in the OGD+IL-10NA group (*p*>0.05) (Fig 6C); the expression of active caspase-3 was increased in the OGD+IL-10NA group but significantly decreased in the OGD+IL-10 group (*p*<0.01, respectively) (Fig 6D). These data indicate that IL-10 up-regulates the expression of p-PI3K and p-AKT and down-regulates the level of active caspase-3 and that these effects can be partly suppressed by IL-10NA.

## Discussion

In the present study, the main findings are as follows: (1) IL-10 reduced apoptosis and inhibited active caspase-3 expression in the cultured rat cortical neurons exposed to OGD; (2) IL-10 protected the neurites in terms of axon length and dendrite number and increased both AKT phosphorylation and GAP-43 expression; (3) LY294002, a specific inhibitor of PI3K/AKT, significantly attenuated the protection from IL-10 in OGD-induced injury. (4) IL-10NA aggravated the OGD-induced injury in the neurons.

As a pleiotropic cytokine, IL-10, which is mainly produced by microglia, plays an anti-inflammatory role in various neuronal injuries and has been documented to play an important neuron-protective role in vivo and in vitro [6,17]. Neurons co-cultured with IL-10 KO glial cells have been found to be more sensitive to damage induced by NMDA or OGD and cerebral infarction volume in IL-10 KO mice is larger than that of the wild type in focal cerebral ischemia [18]. In addition, IL-10 has been documented to directly protect neurons from stroke-induced damage [6,7,18,19]. In the present study, we found that IL-10 dose-dependently increased the survival of neurons S1 Fig, which is in line with the previous study [6]. Furthermore, we used two different methods to determine the apoptosis of neurons. The results showed that IL-10 remarkably decreased the OGD-induced neuronal apoptosis, indicating that IL-10 can protect neurons against OGD-induced apoptosis.

Cytological changes in the injured neurons can appear in many forms, and the most typical changes are manifested in cell soma atrophy, nuclear condensation and DNA fragmentation. However, the structural changes after neuronal injury remain blurred. In Agarwala’s study [20], in the aged rats, 66% of the neurons in the dLGN die after four days of lesion. Several studies have reported dendritic changes in CNS neurons after various in vivo insults [21–24]. In a study [25], neurons experiencing an injury that results in structural changes in their dendrites may retain the potential to survive if the early, injury-induced dendritic degeneration can be blocked from proceeding; an injured neuron, after the loss of its secondary or higher-order dendrites, also loses its ability to communicate with other neurons, which results in neuronal dysfunction or cell death. Dendritic changes often follow the injury immediately, but dendritic alteration is a reversible event in injured neurons under certain conditions. These results indicate that axon and dendrites also play an important role in neuronal survival after the incidence of injury. OGD is widely used as cerebral ischemia injury in vitro [26,27]. We found that OGD led to neuron injury not only in the soma but also in the axon, which is in line with the previous study [28]. Previous studies demonstrated that IL-10 can protect neurons from injury by anti-inflammatory and anti-apoptosis after cerebral ischemia [5,6]. Our work further explores the protective effects of IL-10. We found that the axons in the IL-10 group were longer than those of the OGD group, and approximated those of the normal control group. We also noted that dendrites in the IL-10 group, on average, outnumbered those of the OGD group and that many dendrites of neurons were still maintained after OGD while the axons were obviously shortened. These results suggest that axons are more liable to damage than dendrites, and that IL-10 can protect the neurons against injury by remodeling axons and dendrites.

IL-10 can directly protect cortical neurons by activating AKT and STAT3 signaling pathway [6]. AKT, protein kinase B, is activated by active PI3K by phosphorylating Ser473 and Thr308 sites in AKT amino acid residues [29]. PI3K/AKT signaling pathway participates in the neuronal survival and axon growth [30–33]. As a neuron-specific protein, growth-associated protein (GAP-43) is implicated in axonal growth, plasticity, neuronal differentiation and regeneration [34–37]. Its activities and distributions are regulated by its dynamic interactions with various neuronal proteins [38]. In our present study, IL-10NA reduced the level of p-PI3K phosphorylation (Fig 6B) and increased the expression of active caspase-3 (Fig 6D), neuronal apoptosis (Fig D in S1 File) and the injury of neurites (Fig C in S1 File). In our previous study [10], OGD-induced injury involved BMSCs and the expression of GAP-43 was increased along with growing axons, and located in the soma and axon; BMSCs mainly protected the neurons from injury through the PI3K/AKT/GAP43 signaling pathway. Our results indicate that IL-10 up-regulates the level of GAP-43 by activating AKT and that the effect can be partially inhibited by LY294002. It demonstrates that IL-10 probably protects neurites from injury through the PI3K/AKT/GAP-43 pathway. Caspases belong to the family of cysteine kinase and play a critical role in the regulation of cell apoptosis [39]. Caspase-3, as an executor of apoptosis, can ultimately lead to cell death by activating the fractured factor of DNA [40]. In the diseases involving the central nervous system, such as Alzheimer’s disease and Down’s syndrome, Caspase-3 activation is a key step in the process of apoptotic cascade [33]. In our study, compared with the OGD+IL-10 group, the OGD and OGD+IL-10+LY294002 group had lower expression of pAKT, but higher expression of Caspase-3. This result indicates that IL-10 down-regulates the level of Caspase-3 probably by activating pAKT and the effect can be partially inhibited by LY294002. Taken together, our findings demonstrate that IL-10 probably protects neurons from apoptosis through the PI3K/AKT pathway.

In conclusion, our results support the notion that IL-10 probably protects the neurons from OGD-induced injury through anti-apoptosis and the neurites by activating the PI3K/AKT pathway and down-stream proteins, GAP-43 and Caspase-3. Meanwhile, there are some limitations in our study: for one, we only tested the neuroprotective effect of IL-10 at the cellular level, and more experiments are encouraged to verify the effect at the animal and clinical level; for another, the role of its gene expression in protecting neurites and neuron survival remains unclear. Nevertheless, the present study provides new molecular insight into the neuroprotective effect of IL-10 and suggests its possible therapeutic role in the management of brain ischemia.

## Supporting Information

S1 Fig Viability of neurons measured by MTT after OGD. **p<0.01 vs 0ng/ml, *p<0.05 vs 0ng/ml.
(TIF)

S1 File IL-10NA increased the neuronal apoptosis and the injury of neurites in OGD-induced injury model. a: Normal control group. b: OGD + IL-10 group. c: OGD + IL-10NA (5μg/ml) group. d: OGD group. Data are expressed as mean ± SD (n = 9).*p<0.01 vs OGD+IL-10 group (92.81±6.69), #*p*<0.01 vs OGD+IL10NA group (33.30±10.76), ##*p*<0.01 vs OGD group (65.99±7.61) (Fig C). Data are expressed as mean ± SD (n = 3) *p<0.01 vs OGD+IL-10 group (16.47±2.31), #*p*<0.01 vs OGD+IL10NA group (38.90±2.79), ##*p*<0.01 vs OGD group (26.93±5.45) (Fig D).
(TIF)
