# Supplementary figures and images for: IL-10 Protects Neurites in Oxygen-Glucose-Deprived Cortical Neurons through the PI3K/Akt Pathway
Source: PLoS One. 2015 Sep 14;10(9):e0136959. doi: 10.1371/journal.pone.0136959 (PMC4569574; doi:10.1371/journal.pone.0136959)

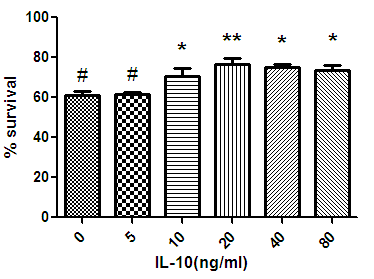

Supplement: S1 Fig — **p<0.01 vs 0ng/ml, *p<0.05 vs 0ng/ml. (TIF) [file pone.0136959.s001.tif]

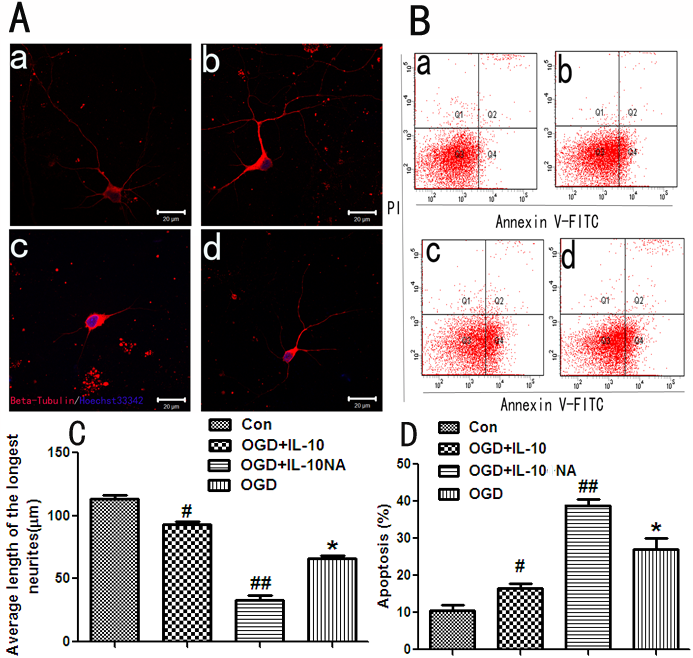

Supplement: S1 File — a: Normal control group. b: OGD + IL-10 group. c: OGD + IL-10NA (5μg/ml) group. d: OGD group. Data are expressed as mean ± SD (n = 9).*p<0.01 vs OGD+IL-10 group (92.81±6.69), #p<0.01 vs OGD+IL10NA group (33.30±10.76), ##p<0.01 vs OGD group (65.99±7.61) (Fig C). Data are expressed as mean ± SD (n = 3) *p<0.01 vs OGD+IL-10 group (16.47±2.31), #p<0.01 vs OGD+IL10NA group (38.90±2.79), ##p<0.01 vs OGD group (26.93±5.45) (Fig D). (TIF) [file pone.0136959.s002.tif]
